# HIV testing and linkage to services for youth

**DOI:** 10.7448/IAS.18.2.19433

**Published:** 2015-02-26

**Authors:** Ann E Kurth, Michelle A Lally, Augustine T Choko, Irene W Inwani, J Dennis Fortenberry

**Affiliations:** 1 College of Nursing and Global Institute of Public Health, New York University, New York, NY, USA; 2 Lifespan Hospital System, Providence, VA, USA; 3 Alpert Medical School, Brown University, Providence, RI, USA; 4 Malawi-Liverpool Wellcome Trust Clinical Research Programme, Blantyre, Malawi; 5 Kenyatta National Hospital, Nairobi, Kenya; 6 Department of Pediatrics, Indiana University, Indianapolis, IN, USA

**Keywords:** HIV testing, HIV continuum of care, youth, adolescents, key populations, development

## Abstract

**Introduction:**

HIV testing is the portal to serostatus knowledge that can empower linkage to care for HIV treatment and HIV prevention. However, young people's access to HIV testing is uneven worldwide. The objective of this paper is to review the context and concerns faced by youth around HIV testing in low- as well as high-income country settings.

**Discussion:**

HIV testing is a critical entry point for primary and secondary prevention as well as care and treatment for young people including key populations of vulnerable youth. We provide a framework for thinking about the role of testing in the continuum of prevention and care for young people. Brief case study examples from Kenya and the US illustrate some of the common barriers and issues involved for young people.

**Conclusions:**

Young people worldwide need more routine access to HIV testing services that effectively address the developmental, socio-political and other issues faced by young women and men.

## Introduction

Youth aged 15–24 represent 39% of new HIV infections in people aged 15 years and older (2012) [1]. Among young people with HIV, most (4 million) live in sub-Saharan Africa [2]. Access to HIV testing and to antiretroviral therapy (ART) for youth remains a concern globally. Young people's HIV testing levels in low- and middle-income countries (LMICs), that contain most global HIV disease burden, is uneven [3]. Fewer than one in five boys and one in three girls aged 15–19 years in Africa report ever HIV testing [2]. The United States has poor sexual health statistics including in HIV testing access [4]; the proportion of US youth who HIV test has remained low at 22% and stagnant since 2005 [5]. For key populations (KPs), including males who have sex with males (MSM), people who inject drugs (PWID), transgender people (TG) and sex workers (SW), access to HIV testing is even more challenging due to marginalization and stigma.

The seek, test, treat, retain and suppress continuum has been promulgated as an approach with potential to bend the curve of the HIV epidemic [6]. Knowledge of serostatus is a starting point for lifesaving ART and to reduce sexual, parenteral, or vertical transmission. The particular HIV testing barriers and facilitators for youth in the HIV continuum of care have had less focus, however. Attention to developmental milestones is critical, e.g., yet most of what is known regarding linkage and retention in care has been based on adult, not youth populations. The sense of invulnerability that many adolescents feel – despite epidemiologic risks – also contributes [7,8]. Young people who are part of KP subgroups face overt discrimination and have lower testing rates than general population youth, facing additional barriers including fear, concerns about confidentiality and cost [4], low self-efficacy [9] and lack of KP-youth-friendly services. In this paper, we highlight critical issues involved for youth, including KPs, along the HIV testing–prevention–treatment continuum.

### Framework: testing as entry to prevention and treatment

**Figure 1:**
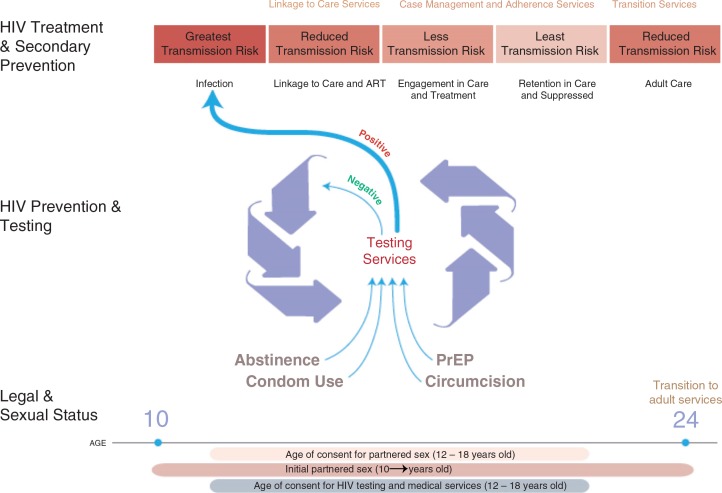
Framework for HIV services for youth in the HIV continuum.

Testing for HIV is offered via provider-initiated testing and counselling (PITC) in health facilities, on a self-initiated basis through voluntary counselling and testing sites (VCT), delivered by staff in homes (home-based or HBCT), through community campaigns and through self-testing. Each modality has benefits and drawbacks specific to youth, yet HIV testing overall serves as a critical core component of the HIV continuum for vulnerable youth. In Figure 1, we graphically summarize a model where testing for HIV functions as a triage portal for needed youth-friendly services around an HIV Continuum of Prevention and Care, including housing, mental health treatment, substance abuse treatment and sexually transmitted infection (STI) services.

At-risk and KP youth [10] need safe opportunities to test, and re-test, for HIV. Acute HIV infection is important to identify [11] and US guidelines call for 4th generation screening tests [12], though these may not be available in many settings globally. Linkage to care for positive youth is especially challenging. Even with dedicated outreach and youth-friendly clinics, only 70% of HIV-infected youth in the US Adolescent Trials Network were successfully linked [13].

The primary goal of HIV care is viral suppression, with strong evidence for immunological advantage from early suppression [14]. Individuals also gain emotional assurance that viral suppression minimizes risk for transmission. This benefit can be realized on a population level [15]. Lifelong ART is challenging, with high attrition post-ART initiation among youth noted in Africa [16] and elsewhere. Technologies that are highly acceptable to young people, such as text messaging, to support adherence are promising [17]. Technology tools also have been used successfully to support HIV testing uptake among adolescents including in US emergency departments [18]. Partner services are critical for HIV-infected youth. Keeping HIV-negative youth healthy and uninfected remains a key goal.

Since the global burden of HIV disease is in sub-Saharan Africa, with two-thirds of all HIV cases and four-fifths of all young persons living with HIV, making new modalities such as self-tests available may help KP youth gain access to serostatus knowledge. In Malawi, in work done by Choko and colleagues, uptake of HIV self-testing among young people aged 16–24 years was consistently higher than among adults aged ≥25 years: 93.7% among those under age 25 versus 65.5% among adults 25 years and older (*p<*0.001). However, only 42.4% of the youth in that study had ever HIV tested, compared with 57.6% of those 25 and older (*p*<0.001) [19], indicating an unmet need as the younger age group is sexually active and exposed to HIV including through sex with older partners more likely to be HIV-infected [20].

In Kenya, where an estimated 100,000 new HIV infections occurred in 2013, girls and KPs are disproportionately affected by HIV [21]. School-based HIV education does not equip youth to seek testing, and there are few youth-friendly facilities available. Policy guidance says minors require parental/guardians’ consent for HIV testing, though Kenya has eliminated age limit as the only criteria. Many healthcare providers are ignorant of this provision, however, and deny unaccompanied adolescents an HIV test. In the country's new HIV roadmap [21], there is commitment to reviewing parental consent for HIV testing for adolescents. Youth who discuss testing with their parents are more likely to HIV test [22]. However, youth often rightfully fear negative reactions from parents and providers, including in schools where they fear isolation and missed opportunities and employment prospects if known to be HIV-positive. In some communities, women cannot give consent without consent by family members (case example, Box 1).

*Box 1*. Benta [a pseudonym], 17 years old, was admitted with her 2 year-old child into the paediatric ward. She comes from a pastoral community, got married at 15 and never attended antenatal clinic. She does not know her HIV status. She and her child are offered provider-initiated HTC. She has to get permission from her mother-in-law who says Benta and her child can be tested but only if the father of the child consents. He cannot be reached by phone, does not visit the family in the hospital for 10 days, and Benta and her child leave hospital without knowing their HIV status.

KPs including MSM and transgender youth in sub-Saharan Africa are often hidden and it is not safe to self-identify to providers (Box 2). Issues faced by youth for HIV testing cut across country contexts or resource boundaries, especially when in persecuted groups like MSM (Box 3 from high-income US setting).

*Box 2*. Paul [a pseudonym] is a transgender youth in Kenya. After high school, he was not able to get a job. One day he dressed like a female and got employed as house help looking after two girls. One day the girls and their mother saw Paul in a mall, dressed as a young man. The mother confronted him claiming Paul was masquerading as a woman with intent to abuse her children. A crowd gathered and physically assaulted Paul. Police officers forcibly took him to a nearby clinic where he was tested for STIs including HIV without consent. Results were disclosed without consent. He lost his job and had to re-locate.

*Box 3*. Michael [a pseudonym] is an 18-year-old who has been homeless for 12 months. He exchanges sex with other men for money in order to survive, and most do not use condoms. He was recently tested for HIV, and he was told it was positive. He is sure it was a mistake, and avoids going to any health facility.

## Discussion

### Developmental issues relevant to HIV testing

The dividing line of adolescence and adulthood is often seen as a sharp transition (e.g. at 18 or 21 years). These age transitions mark relevant thresholds for age of consent for HIV testing, for HIV medical services, and for partnered sex (Figure 1). Although adolescence is developmentally continuous and subject to substantial individual, cultural and national variation, it is useful to think of the HIV service continuum in the context of early (10–14 years), middle (15–17 years) and late adolescence (18 years and older).

#### Early adolescence (10–14 years)

Early adolescence is marked by puberty, achievement of adult size and gender-typical body contours with new assumptions about responsibility for sexual behaviour. Family and economic situations may require contributions to household income and sibling care that affect schooling and vocational opportunities [23]. Puberty in many cultures is associated with initiation rites that may not include HIV prevention messages [24–26] and that carry potential risks including non-medical circumcision [27,28]. (Voluntary medical male circumcision plays a critical role in HIV prevention and is well-accepted by many young men and parents [29].)

Development of sexual orientation is a key task. Often heterosexual identity is assumed as the “normal” outcome while other identities may be considered deviant [30]. During early and middle adolescence, sexual orientation has substantial variation and fluidity and often, lack of congruence between identity and behaviour [31].

Much emphasis is given to timing of coitus [32,33]. Over-emphasis on adolescent coitus complicates appropriate matching of services because many adolescents do not have coitus yet engage in other partnered sexual behaviours associated with HIV risk. Same-sex partnered behaviours often are omitted from sexuality education or relegated to being entirely risky without contribution to sexual or relationship satisfaction.

As pointed out in Figure 1, there may be a mismatch and delay between when young people begin having sex and when they can legally obtain HIV testing independently. HIV tests before first partnered sexual event have unproven benefits (e.g. normalization of testing) and harms (e.g. false security). After first partnered sex, it is unclear when young people begin to seek HIV testing on their own, or when clinicians recommend testing [33], despite guidelines that paediatricians and youth providers offer on HIV testing around age 13 onward [7].

Health providers should ask about sexual activity among younger patients. Many youth are not consensually sexually active and may acquire HIV via sexual abuse, or may have acquired HIV perinatally. In both these cases the young person may not be willing to share their sexual activity history at their first encounter with a new provider. Adolescents’ non-consensual sexual experiences and intimate partner violence (IPV) may increase risky behaviours [34,35]. Few IPV victims report discussions with a provider, demonstrating the importance of routine assessment for partner violence [36].

Sexuality education may occur in secondary schools, although content varies greatly [37–39] and this misses out-of-school youth. Primary emphasis on abstinence-until-marriage is less effective for HIV prevention than age-appropriate, comprehensive programs [40–42]. Informal sources of information including social media are ubiquitous in adolescents’ daily lives worldwide [32,43–45].

#### Middle adolescence (15–17 years)

By this stage some functional competencies needed to manage one's health may be in place. However, many adolescents lack skills or status to negotiate complex systems [46].

Adolescents’ participation in the HIV continuum of care as consumers of health products (e.g. condoms, pregnancy tests) is infrequently explored. Sale of HIV self-testing kits is not age-restricted although costs, test implementation fidelity and point-of-sale confidentiality have not been fully explored with young, high-risk persons [47–49] . Early data on self-testing acceptability, as seen in the Malawi example, are encouraging.

Many youth in this age group routinely have sex, especially in subgroups where survival depends on sexual exchange, which is often unprotected given power differentials in these encounters. Many KP youth live on their own, though are not yet an age of legal majority.

#### Late adolescence – youth (18–24 years)

Age 18 often is considered adulthood; however, it is now known if significant brain development including in the prefrontal cortex responsible for decision-making does not actually mature fully until age 25, which may influence vulnerability and resilience of young people [50] in terms of HIV risk and testing decisions.

### Legal issues and the HIV continuum of care for adolescents

Three highly variable (from jurisdiction to jurisdiction) milestones dictate legal thresholds for adolescents’ engagement in the HIV continuum of care: age of consent for partnered sex; for HIV testing; and, for HIV medical services (Figure 1). Adolescents’ differential legal access to HIV-related testing and other services is based on traditional assumptions of parental rights as well as restricted autonomy of children [51].

Identification of sexual activity of minors less than the age of consent threshold may mandate reporting to child protection authorities [52]. Given concerns about widespread victimization and HIV (especially of girls and younger adolescents) [53], some countries have enacted “defilement” laws that can be enforced without regard for consensuality of the partnered sex [54].

The ethical concept of “the mature minor,” while infrequently given legal sanction for adolescents’ self-consent for general medical treatment [55], informs legal exceptions to consent requirements for HIV [56,57]. Age thresholds for consent of diagnostic HIV testing are widely variable, often as young as 12 years of age. These laws recognize that parental permission is a critical barrier to HIV testing, and could invoke physical danger if non-marital or same-sex activity is suspected or disclosed. Age thresholds for minor self-consent for HIV is sometimes addressed within the context of laws that allow for STI assessment [58,59]. However, medical HIV treatments are lifelong and expensive, requiring ongoing relationships with providers [60].

#### Cross-cutting issues for youth that affect HIV testing uptake

Across all age groups, stigma adversely affects each phase of adolescents’ engagement with the HIV continuum [61]. Internalized stigma may particularly effect HIV testing behaviours while anticipated stigma may have especially strong effects on care-seeking and adherence [58,59].

Physical, sexual and emotional aggression is experienced by many youth, especially in KP groups (e.g. sexual minorities), where microaggressions also have a damaging impact [62]. Legal protection, and campaigns to reduce bullying and other forms of aggression, are needed. Finally, many young people are economically disadvantaged relative to adults and cost barriers to HIV testing must be effectively addressed.

### Solutions to increase HIV testing uptake among youth including KPs

HIV testing services must be available to all young people, particularly those from KPs. Health literacy is an issue for many adolescents [63] which “youth-friendly” programs may address [64]. Demand creation strategies have been used effectively for HIV testing via social marketing campaigns [65] and should be further employed. Testing availability where youth gather, and user-friendly free or subsidized test kits, may increase uptake. Once confirmed positive, linkage to care is critical and youth should access treatment services in whatever clinical venue is preferred, whether paediatric or adult (to reduce loss to follow-up when forced into adult services at arbitrary age cutoffs like 18 years). Testing and care are enhanced by respectful health care teams, and reduction of resource barriers such as transport fees and homelessness, that disrupt treatment continuity. Counselling can address the utility of ART taking into account developmental stages in which many young people may feel invulnerable and find navigating complex health systems overwhelming, especially in the commonly-occurring context of depression, substance use and other co-morbidities. Failure to link/retain adequately has dire consequence; Zanoni and Mayer estimate that only 6% of HIV-positive US youth are virally suppressed. They recommend that HIV testing be integrated wherever youth interact with health systems, as well as in youth venues, to normalize and promote testing and recurrent testing among high-risk and KP youth [66].

## Conclusions

### Recommendations and research gaps

Adolescents and young adults worldwide deserve better access to HIV testing and re-testing. We recommend that testing venues be made more youth-friendly, and promising new approaches like self-testing be monitored regarding how well they work for youth. Implementation science can identify optimal ways to improve HIV testing access and delivery for youth [67]. HIV testing in prevention of maternal to child transmission (PMTCT), antepartum care and voluntary medical male circumcision (VMMC) campaigns alone is insufficient. For youth, HIV testing is a key portal for linkage to necessary HIV care and prevention services.

Despite international and national guidelines, HIV testing for adolescents is still not consistently done in high- [68] or lower-income countries. Providers worldwide [69] must consistently assess sexual behaviours or partnership risks, so that appropriate counselling based on the young person's actual needs is not pre-empted [10]. HIV testing can be made more youth-friendly [70] even under the constraints of ART scale-up [71], but truly supportive services ultimately must rely on empathetic, self-aware [72] and professional health provider behaviours [73] including assurance of confidentiality around test results [74], reinforcement for those testing HIV-negative, and social and clinical support for those testing HIV-positive. Ensuring youth rights cannot occur only within clinic walls but must extend to the community and to social as well as legal norms [75].

There are social justice and public health imperatives to focus on structural factors that keep young people from freely HIV testing – including laws that harm KPs and program structures and costs that restrict access. The HIV Investment Framework points out that contraceptive services are a cost-effective portal for youth HIV testing [76], of even more importance in LMIC settings where a higher proportion of the population are of reproductive ages [77]. Achieving universal access to youth-friendly services worldwide would cost around US$ 1 per adolescent [78]. Program quality monitoring of HIV testing access [79], and implementation of best HIV testing practices, for young people must a part of the HIV agenda if we are to achieve generations with fewer HIV infections and provide better care of those living with HIV.
